# Optimizing Microneutralization and IFN-γ ELISPOT Assays to Evaluate Mpox Immunity

**DOI:** 10.3390/vaccines13010027

**Published:** 2024-12-31

**Authors:** Yinyi Yu, Krystal Meza, Chase Colbert, Daniel F. Hoft, Anna Jaunarajs, Azra Blazevic, Sharon E. Frey, Getahun Abate

**Affiliations:** 1 Division of Infectious Diseases, Allergy and Immunology, Saint Louis University, St. Louis, MO 63104, USAdaniel.hoft@health.slu.edu (D.F.H.); azra.blazevic@health.slu.edu (A.B.); sharon.frey@health.slu.edu (S.E.F.); 2 EMMES, Rockville, MD 20850, USA

**Keywords:** mpox, vaccinia, FRNT, microneutralization, PRNT, IFN-γ ELISPOT

## Abstract

Background: Available assays to measure pox virus neutralizing antibody titers are laborious and take up to 5 days. In addition, assays to measure T cell responses require the use of specific antigens, which may not be the same for all pox viruses. This study reports the development of robust assays for the measurement of mpox-specific neutralizing antibodies and IFN-γ-producing T-cell responses. Methods: Fourteen samples from 7 volunteers who received Modified Vaccinia Ankara-Bavarian Nordic (MVA-BN) were used. The focused reduction neutralization test (FRNT) was performed using the mpox-specific A29 monoclonal antibody. Optimization and further development of FRNT were conducted using the plaque reduction neutralization test (PRNT) as the gold standard. The mpox-specific IFN-γ ELISPOT assay was optimized using different mpox antigen preparations. Results with pre-vaccination samples were compared with post-vaccination samples using the Wilcoxon matched-pairs test. Results: Pre-vaccination and post-vaccination sera (*n* = 7) had FRNT50 (i.e., titers that inhibited at least 50% of the virus) of 109.1 ± 161.8 and 303.7 ± 402.8 (mean ± SD), respectively. Regression analysis of fold changes in FRNT50 and PRNT50 showed that the two assays closely agree (*n* = 25 tests on paired samples, R^2^ of 0.787). Using UV-inactivated mpox as an antigen, the number of IFN-γ spot-forming T cells (SFC) in pre-vaccination samples (16.13 ± 15.86, mean ± SD) was significantly lower than SFC in post-vaccination samples (172.9 ± 313.3, mean ± SD) with *p* = 0.0078. Conclusions: Our newly developed microneutralization test has a good correlation with PRNT. UV-inactivated mpox is an appropriate antigen for the ELISPOT assay that measures mpox cross-reactive T cells. These assays will be useful in future mpox vaccine studies.

## 1. Introduction

Mpox virus, a member of the Poxviridae family along with smallpox and cowpox viruses, is the etiologic agent of zoonotic disease [1]. Mpox has two clades, each with two subclades [2,3]. In animals, clade I is more virulent than clade II [2,3]. Mpox has caused major outbreaks in Africa and around the world [4,5,6,7,8]. The recent multi-country outbreak that started in May 2022 was caused by clade IIb, involved more than 118 countries (or territories), and was declared a public health emergency by the World Health Organization (WHO). As of September 2024, 109,699 cases were confirmed, and there were 236 deaths [9]. This major outbreak with clade IIb is largely under control, but low-level transmission continues to occur. In the last year, between 1 October 2023 and 20 September 2024, there were 5476 cases from North and South America and 1986 cases from the Western Pacific Region [9]. In addition, an mpox outbreak with the clade I strain has emerged in Africa and is spreading in the Democratic Republic of the Congo (DRC) and other African countries [9,10]. In 2024, as of 27 October, 10,722 confirmed cases have been reported from 19 African countries: 8607 from DRC, 1509 from Burundi, and 220 from Uganda [9]. It is believed that the number of cases in Africa could be much higher, as many cases remain untested due to the limited availability of confirmatory tests [9].

Immunization is an essential strategy to control mpox outbreaks. The use of MVA-BN for pre- and post-exposure vaccination of high-risk populations has helped control the major global mpox outbreak. However, low-level transmission is still a problem. Measuring mpox humoral and cellular immunity induced by vaccines or natural infection requires robust assays. The plaque reduction neutralization test (PRNT) is one of the commonly used assays to measure mpox-neutralizing antibody titers [11,12,13]. PRNT has been a gold standard to measure vaccinia and mpox neutralizing antibody titers following vaccination or infection in humans and nonhuman primates [11,12,13]. However, PRNT has some disadvantages. First, in PRNT, neutralizing antibody titers are quantified manually by comparing virus-induced plaques in cell cultures containing a sera-virus mixture with cultures containing no sera [14]. Therefore, the readout is prone to variation. Second, PRNT suffers from a long turnaround time, taking 2–5 days to obtain results [14,15,16]. Third, the test is laborious and is conducted in 24-well plates, requiring larger volumes of sera, virus suspension, and media. Assays that will be performed in 96-well plate formats, such as focused reduction neutralization test (FRNT), have the potential to save resources and allow the use of a more sensitive method of virus detection to shorten the turnaround time.

Mpox virus, like other pox viruses, replicates in the cytoplasm of host cells and produces two types of infectious forms: extracellular enveloped virus (EEV) and intracellular mature virus (IMV) [17,18]. The IMV is released during cell lysis, whereas the EEV, which has a lipid membrane wrapped around the IMV, is released through exocytosis [18]. The IMV form expresses proteins such as A29, M1R, E8L, and L1R [19]. A29 with M1R and B6R proteins has shown promise as a vaccine candidate [20]. Antibodies against mpox A29 and the corresponding vaccinia A27 proteins have been successfully used in enzyme-linked immunosorbent assays to measure antibody responses following mpox infection [21] and could be the best targets for developing a new FRNT.

A commonly used assay for T cell responses is the IFN-γ ELISPOT, but to our knowledge, there are no studies that have reported optimization of antigens and other important conditions for the use of this assay to measure mpox-specific T cell immunity. Therefore, this study was carried out with the objectives of optimizing two commonly used assays, FRNT, also called microneutralization, and the IFN-γ ELISPOT assay using mpox virus. FRNT was optimized using the plaque reduction neutralization test (PRNT) as a reference, and mpox IFN-γ ELISPOT was optimized using vaccinia as a control.

## 2. Materials and Methods

Specimen: Peripheral blood mononuclear cells (PBMC) and sera were collected from 7 individuals before and after vaccination with MVA. The use of human samples for this project was approved by the Institutional Review Board (Approval Number 33195). Blood was collected in CPT (Becton Dickinson, Franklin Lakes, NJ, USA) and red cap (Becton Dickinson, Franklin Lakes, NJ, USA) tubes for PBMC and sera separation, respectively. CPT tubes are centrifuged at 2750 rotations per minute (rpm) for 25 min at room temperature; the cell layer is transferred to 50 mL plastic conical tubes, washed three times by resuspending in RPMI medium with 1% fetal bovine serum (FBS, ThermoFisher Scientific, Berkeley, MO, USA), and centrifuging at 1260 rpm for 10 min. PBMC cell counts were made from the final tube, and 1 × 10^7^ cells resuspended in 5% glucose and 20% DMSO in FBS were aliquoted into appropriately labeled cryovials. Aliquoted PBMCs were stored in liquid nitrogen. For separation of sera, blood in red cap tubes was left at room temperature for about 1 h and centrifuged at 2700 rpm for 15 min at room temperature, and sera were aliquoted into labeled Sarstedt tubes and frozen at −80 °C. Each aliquot of sera is heat-inactivated by placing it in a 56 °C water bath for 30 min before use.

Pre- and post-MVA-BN vaccination samples from 7 volunteers were tested multiple times during assay development. Three volunteers with a history of prior smallpox vaccination received one dose, and four volunteers who were vaccinia-naïve received two doses of MVA-BN four weeks apart. Sera and peripheral blood mononuclear cells (PBMC) were obtained from all volunteers before and 14 days after full vaccination with MVA-BN.

Virus stock preparation: Clade IIb mpox culture (NR-2500, Bei resources, Bethesda, MD, USA) was conducted in a BSL-3 laboratory, and vaccinia (Dryvax) culture was conducted in a BSL-2 laboratory. BSC40 cell lines (ATCC, Manassas, VA, USA) are cultured in T-150 or T-75 flasks (Corning, San Diego, CA, USA), and as soon as the cell monolayer is confluent, the medium is removed, and virus suspension is just enough to cover the monolayer is added. The plates were kept in a biological safety cabinet at room temperature for 60 min to allow virus adsorption with gentle rocking of flasks every 15 min. Then, the serum-free minimum essential medium was added (i.e., 20 mL for T-150 or 10 mL for T-75 flasks), and the flasks were incubated at 37 °C and checked for cytopathic effects daily. When the cytopathic effect was evident in ≥75% of the cell layer (i.e., usually day 3), virus cultures were harvested. To harvest viral cultures, flasks kept in a plastic tub were placed flat in a freezer. After the flasks were completely frozen, they were thawed in a biological safety cabinet at room temperature. As soon as the medium began to thaw, flasks were tapped gently against the palm in order that sheets of ice help remove the cells from the plastic. Virus cultures were pooled, sonicated in a water bath sonicator for 30 s twice, centrifuged at 1260 rpm to pellet residual cells and cell debris, and supernatants were aliquoted into appropriately labeled 2 mL Sarstedt tubes with O-rings (ThermoFischer Scientific, Berkeley, MO, USA). Viral number was quantified by culturing different dilutions of the virus from 3–5 aliquots in BSC40 cell cultures. The number of plaque-forming units (PFU) on day 3 or 4 of culture in different dilutions allowed the determination of PFU/mL of aliquoted stocks.

Inactivation of mpox virus: For some experiments, mpox virus was heat- or UV-inactivated. For UV inactivation, psoralen was added to virus aliquots with known PFU/mL at a final concentration of 10 µg/mL. The psoralen virus mixture was vortexed and incubated at room temperature for 10 min. The virus suspension was then transferred into a 55 cm^2^ mL Petri dish with no more than 10 mL per Petri dish, exposed to long-wave UV light at a liquid-to-bulb distance of about 7 inches for 8 min with gentle shaking every 2 min. Heat-inactivated mpox was generated by keeping vials containing live mpox in a 60 °C water bath for 15 min. UV- and heat-inactivations were confirmed by a plaque assay of undiluted virus aliquots. Inactivated mpox did not cause plaque formation.

PRNT: PRNT was performed as described previously [11,22,23]. Briefly, sera were heat-inactivated by placing thawed sera into a 56 °C water bath for 30 min. Sera dilutions ranging from 1 in 4 to 1 in 4096 were used. Sera dilutions were prepared in DMEM (Sigma, Saint Louis, MO, USA) media containing 100 IU penicillin and 100 µg/mL streptomycin and incubated with approximately 100 plaque-forming units (PFU) of mpox virus for 24 h at 37 °C in 5% CO_2_. Following incubation, the sera were added to duplicate wells of a 24-well tissue culture plate containing a confluent monolayer of BSC40 cells and incubated for 48 h at 37 °C with 5% CO_2_. Monolayers were stained with crystal violet in order to visualize plaques, and the PRNT50 neutralizing titer was determined as the dilution, resulting in a 50% reduction in total plaques.

FRNT: FRNT with live mpox was conducted in 96-well plates. Sera heat-inactivation and serial dilutions were similar to PRNT. FRNT was optimized by testing different numbers of BSC40 cells, different PFUs, different dilutions of primary antibody, and different incubation periods of the virus–serum mixture. BSC40 cells were cultured in 96-well plates at a concentration of 2 × 10^5^, 6 × 10^5^, 8 × 10^5^, or 1 × 10^6^ per well. Different mpox dilutions (i.e., 15 PFU, 30 PFU, 60 PFU, or 150 PFU) were added to wells containing different concentrations of BSC40 cells, and cultures were incubated at 37 °C with 5% CO_2_ for 24 h. Two dilutions of (i.e., 1 in 2500 and 1 in 5000) of primary antibody (Mpox A29 antibody, Sino Biological, Wayne, PA, USA) were used to identify focus-forming units (FFU). A secondary antibody (goat anti-mouse antibody, Invitrogen, Carlsbad, CA, USA) was used in a 1 in 2000 dilution. Conditions that could identify FFU better were selected to test samples at pre- and post-vaccination time points. Briefly, in the optimized assay, different dilutions of sera were mixed with an equal volume of virus suspension with an estimated 150 PFU/well and incubated overnight (8–10 h) at 37 °C. The serum–virus mixture was added to 96-well plates containing nearly confluent BSC40 cells. After 1 h incubation with BSC40 cells at 37 °C for 1 h, 1–2 drops of methylcellulose in 2% FBS were added into each well, and the plate was incubated at 37 °C for 24 h. Then, cells were fixed in 5% paraformaldehyde for 30–60 min and washed twice in PBS and once more in focus-forming assay wash buffer (i.e., PBS + 0.005% Triton X-100 (*v*/*v*)). Primary mouse A29 monoclonal antibody (mAb; Sino Biological) solution in FFA buffer was added to each well and incubated at 4 °C overnight, followed by washing three times in FFA buffer. A horseradish peroxidase-labeled secondary anti-mouse antibody (Invitrogen) solution in FFA buffer was added, and the plate was incubated at room temperature for 2–3 h. The plate was washed, peroxidase substrate (Trublue, Sigma, Saint Louis, MO, USA) was added, and it was incubated for 15–20 min before the wells were rinsed with Millipore water, and spots were read using a CTL ImmunoSpot analyzer. The reagents necessary for this assay are listed in Supplementary Table S1. Data were considered interpretable if the average FFU in wells with virus alone ranged from 15 to 50, positive control serum titers were ≥16, and negative control titers were <4.

IFN-γ ELISPOT: IFN-γ ELISPOT kit was obtained from BD Bioscience (Franklin Lakes, NJ, USA), and the assay was performed according to the manufacturer’s instructions. Briefly, an anti-IFN-γ capture antibody in coating buffer was added to each well of a 96-well plate and incubated at 4 °C overnight. Plates were washed once in RPMI, and 200 µL of RPMI with 10% FBS was added to each well and incubated for 2–4 h at 37 °C before adding different numbers of PBMC (3 × 10^5^ or 5 × 10^5^ PBMC) into each well, followed by antigens such as mpox, vaccinia, or phorbol 12-myristate 13-acetate (PMA, Sigma, Saint Louis, MO, USA). PMA at 10 µg/mL was used as a positive control, and the medium alone was used as a negative control. Tests were performed in triplicates. Then, the plates were incubated for 24 h at 37 °C in a 5% CO_2_ incubator. Then, the plate was washed twice in water and three times in PBS + 0.05% Tween 20 (*v*/*v*) (PBST) with 3 min incubation at room temperature between each wash. Biotin-conjugated anti-IFN-γ was added to each well and incubated for 2 h at room temperature, followed by washing with PBST three times and the addition of streptavidin-horseradish peroxidase solution. Then, the plates were incubated for 1 h at room temperature, washed four times, and a developing buffer (i.e., 15 mL of 0.1 M sodium acetate with 4 µL of 30% H_2_O_2_ and 500 µL of 10 mg/mL solution of 3-amino-9-ethylcarbazole) was added. After 10–30 min of incubation at room temperature, the color reaction was terminated by washing plates 3 to 4 times in water; plates and lids were immersed in 5% formalin solution for 1 h for decontamination prior to removing from the BSL-3 facility, washed, and spots counted using the Immunospot Micro Analyzer. Mpox and vaccinia antigen preparations (i.e., live, heat-inactivated, and UV-inactivated), the concentration of antigens, and cell concentration per well were optimized. Reagents necessary for IFN-γ EISPOT are listed in Supplementary Table S2.

Statistical analysis: The highest serum dilution that reduces focus-forming units or plaques by 50% (FRNT50 or PRNT50) was identified. The results from FRNT50 and PRNT50 were compared with FRNT60 and PRNT60 results. The Wilcoxon matched-pairs test was used to compare FRNT, PRNT, and IFN-γ ELISPOT results on pre-vaccination and post-vaccination samples. Regression analyses were used to compare fold titer changes obtained by FRNT and PRNT.

## 3. Results

### 3.1. Effects of Number of BSC40 Cells, PFU, and Dilution of Primary Antibody in FRNT

**Figure 1:**
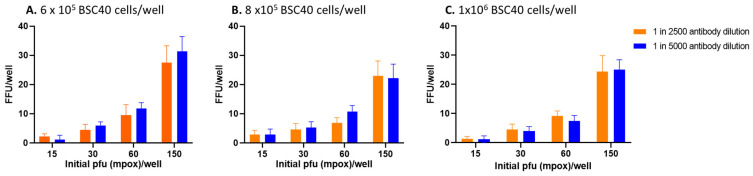
Optimizing primary antibody concentrations, numbers of BSC40 cells, and mpox viral numbers for use in FRNT. BSC40 cells were cultured in 96-well plates at a concentration of 2 × 10^5^, 6 × 10^5^, 8 × 10^5,^ and 1 × 10^6^ per well. Mpox dilutions ranging from 15 PFU to 150 PFU per well were added to wells, and cultures were incubated at 37 °C with 5% CO_2_ for 24 h. Two dilutions of (i.e., 1 in 2500 and 1 in 5000) of primary antibody were used in FRNT. BSC40 cell number per well did not affect FFU if the viral dilution added to the wells provided 3000 PFU/mL, which is a working viral suspension. A viral dilution that gives 3000 PFU/well gives >20 FFU. BSC40 cell numbers as low as 2 × 10^5^/well provided similar results.

Figure 1 shows that the use of 150 PFU/well provided the highest focus-forming units (FFU) per well with all tested concentrations of BSC40 cells and both dilutions of anti-A29 monoclonal antibody used for the detection of spots. After 24 h of incubation at 37 °C, a BSC40 cell concentration that gives confluence, 150 PFU of virus, and 1 in 5000 antibody dilution were selected for further study.

### 3.2. Further Testing of Selected Parameters for FRNT

**Figure 2:**
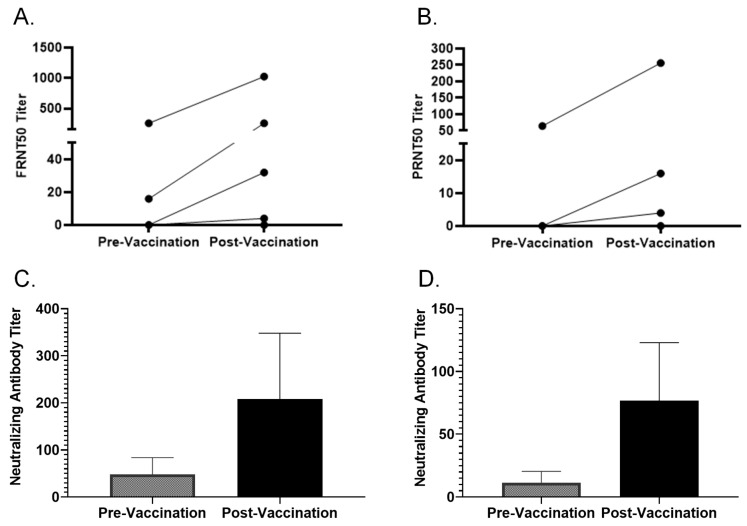
Mpox neutralizing antibody titers were measured by FRNT and PRNT (*n* = 7). (**A**,**B**) show post-vaccination changes in FRNT50 and PRNT50 results, respectively. FRNT50 and PRNT50 correlated with Pearson r = 0.995 for pre-vaccination results (*p* < 0.0001, 95% CI 0.9926–1.0) and Pearson r = 0.9637 for post-vaccination results (*p* < 0.0082, 95% CI 0.5442–0.9977). (**C**,**D**) show actual neutralization titers (i.e., without any cut-off to interpret results) in pre- and post-vaccination samples measured by FRNT and PRNT, respectively. Neutralization titers for pre- and post-vaccination samples were 48 ± 35.78 (mean ± SE) and 207.4 ± 140.6 (mean ± SE) when measured by FRNT. Neutralization titers for pre- and post-vaccination samples were 11.43 ± 9.05 (mean ± SE) and 76.57 ± 46.37 (mean ± SE) when measured by PRNT.

FRNT was compared with the already established PRNT using pre- and post-vaccination samples from 7 volunteers who received two doses of MVA-BN (*n* = 4) or a booster (*n* = 3). Figure 2 shows both pre- and post-vaccination FRNT50 and PRNT50 titers. The pre-vaccination FRNT50 titers were 0, 0, 16, 0, 256, 64, and 0, and the corresponding PRNT50 were 0, 0, 0, 0, 64, 16, and 0 for 7 volunteers. Three samples with high pre-vaccination titers were from volunteers with a history of prior smallpox vaccination. The post-vaccination FRNT50 titers were 32, 0, 256, 4, 1024, 128, and 8, whereas the post-vaccination PRNT50 were 16, 0, 0, 4, 256, 256, and 4. The FRNT plate picture is shown in Supplementary Figure S1.

### 3.3. Comparing Different Cut-off Values to Interpret FRNT and PRNT Results

PRNT was used as a gold standard because it was the assay we and other Vaccine and Treatment Evaluation Units in the US had used consistently in smallpox vaccination trials [13,14,23,24,25,26,27,28]. Different cut-offs in the reduction in vaccinia-specific PFU units have been used to interpret the results of PRNT. A 50% neutralization titer (PRNT50) is defined as the highest serum dilution that shows at least 50% plaque reduction [29]. We compared 50%, 60%, and 90% reductions in FFU or PFU as a cut-off to measure the mpox-neutralizing antibody titers obtained by FRNT and PRNT. FRNT50 and PRNT50 results are shown in Figure 2A,B. FRNT50 and PRNT50 correlated with Pearson r = 0.995 for pre-vaccination results (*p* < 0.0001, 95% CI 0.9926–1.0) and Pearson r = 0.9637 for post-vaccination results (*p* < 0.0082, 95% CI 0.5442–0.9977). A similar correlation was obtained when PRNT60 was used. All pre- and post-vaccination sera had PRNT90 titers < 4. Actual neutralization titer values in pre- and post-vaccination samples measured by FRNT and PRNT without any cut-off are shown in Figure 2C and Figure 2D, respectively. Neutralization titers for pre-vaccination samples were 48 ± 35.78 (mean ± SE) and 11.43 ± 9.05 (mean ± SE) when measured by FRNT and PRNT, respectively. Neutralization titers for post-vaccination samples were 207.4 ± 140.6 (mean ± SE) and 76.57 ± 46.37 (mean ± SE) when measured by FRNT and PRNT, respectively.

### 3.4. FRNT and PRNT on Multiple Paired Samples

**Figure 3:**
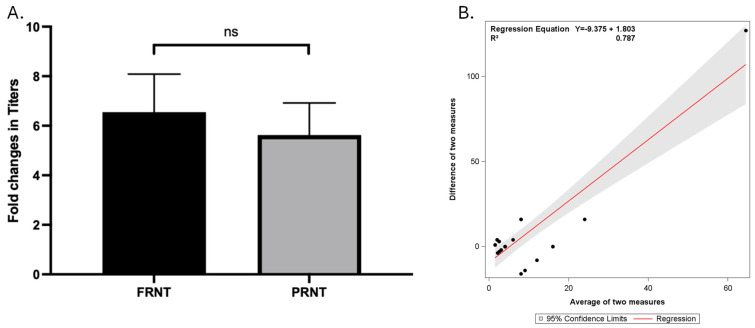
Comparison of all fold changes in mpox neutralizing antibody titer obtained by FRNT and PRNT (total 25 test results from FRNT performed in parallel with PRNT). (**A**) shows fold changes in mpox neutralizing antibody titers obtained by FRNT and PRNT. Seven paired (pre- and post-vaccination) samples were tested multiple times by FRNT and PRNT in parallel. The fold changes of 25 total tests were used for analysis. Fold differences were calculated by dividing post-vaccination titer by the corresponding pre-vaccination titer. For samples with pre-vaccination titer below the lowest dilution in the assay, a value of 1 is entered. Fold changes in the titer of mpox-neutralizing antibodies obtained by FRNT and PRNT were 11.41 ± 5.08 (mean ± SE) and 5.44 ± 1.25, respectively. The fold changes in neutralizing antibody titers measured by the two methods were not significantly different (*p* = 0.25, Wilcoxon match-pair test). (**B**) Regression analysis of the difference between the fold change in the two assays (FRNT-PRNT) and the average of the fold change in the two assays. The slope of the regression line is non-zero (slope = 1.803) with an R-squared of 0.787, suggesting a positive linear relationship between the difference between the two measures and the average of the two measures. As the average fold change in the two assays increases, the difference between the fold change in the two assays appears to increase at a slightly higher rate. ns, not significant.

We tested seven paired samples multiple times with FRNT and PRNT. Four paired samples were tested three times, two paired samples were tested four times, and one paired sample was tested five times. Laboratory personnel were blinded. Figure 3 summarizes all the mpox-neutralizing antibody titer results. Figure 3A shows that fold changes in the titer of mpox-neutralizing antibodies obtained by FRNT and PRNT were 11.41 ± 5.08 (mean ± SE) and 5.44 ± 1.25, respectively. The fold changes in neutralizing antibody titers measured by the two methods were not significantly different (*p* = 0.25, Wilcoxon match-pair test). Regression analysis (Figure 3B) showed that there was a positive linear relationship between the difference between the two measures and the average of the two measures (R^2^ of 0.787).

### 3.5. Effects of Number of PBMC, Antigen Preparations, and Concentration of Antigens on IFN-γ ELISPOT Results

**Figure 4:**
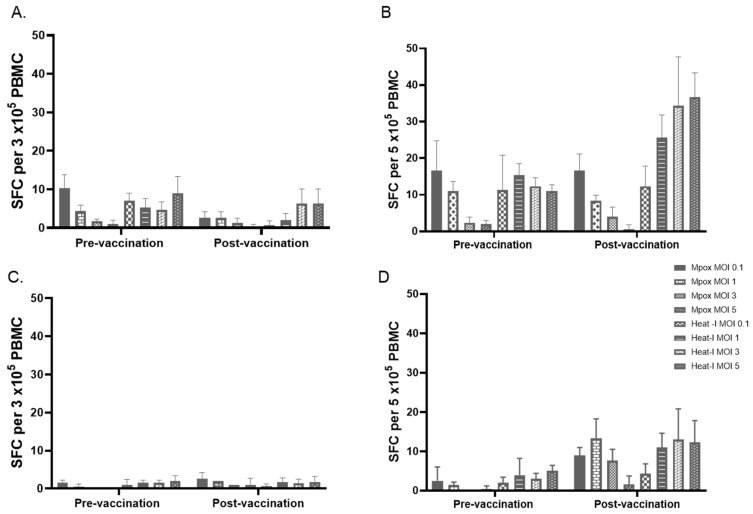
Testing IFN-γ ELISPOT with different PBMC concentrations and different Mpox antigens. Live mpox virus and heat-inactivated mpox at multiplicity of infections (MOI) ranging from 0.1 to 5 were used. (**A**,**C**) show results on pre- and post-vaccination samples of volunteers 1 and 2, respectively, tested with a PBMC concentration of 3 × 10^5^/well. (**B**,**D**) show results on pre- and post-vaccination samples of volunteers 1 and 2, respectively, tested with a PBMC concentration of 5 × 10^5^/well. Heat-inactivated mpox was generated by incubating mpox in a 65 °C water bath for 15 min. Inactivated virus formed no plaques. Live mpox and heat-inactivated mpox did not induce IFN-γ SFC at a cell concentration of 3 × 10^5^ per well and tested concentrations of antigens. Medium-rested controls had SFC of 15.5 ± 11 (mean ± SD) for volunteer 1 and 17 ± 12 for volunteer 2. PMA-stimulated positive control cultures of volunteers 1 and 2 had SFCs of 731 ± 190 and 783 ± 31 (mean ± SD), respectively. Volunteer 1 had a good number of SFC with heat-inactivated mpox (MOIs 1, 3, and 5) when 5 × 10^5^ PBMC per well is used.

**Figure 5:**
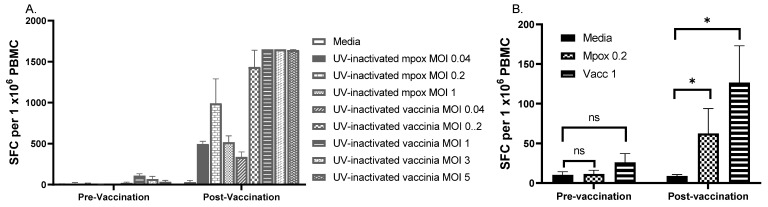
IFN-γ ELISPOT using UV-inactivated mpox and vaccinia as antigens. (**A**) shows SFC after stimulation of pre- and post-vaccination PBMC from one volunteer tested in triplicates with different concentrations of UV-inactivated mpox and vaccinia. Three MOIs of mpox (MOI 0.04, 0.02, and 1) and three MOIs of vaccinia (MOI 0.04, 0.2, and 1) were tested on two additional volunteers with similar results. UV-inactivated virus formed no plaques in a plaque assay. UV-inactivated mpox at MOI of 0.2 and UV-inactivated vaccinia at MOI of 1 induced the optimal number of SFC. (**B**) shows ELISPOT results on seven paired samples using selected concentrations of UV-inactivated mpox (MOI 0.2) and UV-inactivated vaccinia (MOI 1). The number of SFC in pre-vaccination PBMC stimulated with mpox or vaccine was not different from SFC in medium-rested cells with *p* = 0.87 and 0.22, respectively. The number of SFC in post-vaccination PBMC stimulated with mpox or vaccinia was significantly higher compared to SFC in the medium-rested control with *p* = 0.03 (Wilcoxon-matched pairs test). ns, not significant. * *p* < 0.05.

Figure 4 shows results on pre- and post-vaccination PBMC from two volunteers who received two doses of MVA. Figure 4A and Figure 4B show results obtained after stimulation of 3 × 10^5^ PBMC and 5 × 10^5^ PBMC, respectively, with live mpox or heat-inactivated mpox for 24 h at different MOIs. The results showed that post-vaccination PBMC from one of the volunteers had a marked increase in the number of SFC after stimulating 5 × 10^5^ PBMC per well with heat-inactivated mpox at MOIs 1, 3, and 5 (Figure 4B). The need to have a PBMC concentration of 5 × 10^5^ per well may make IFN-γ ELISPOT less applicable on precious clinical trial samples. Therefore, we tested pre- and post-vaccination PBMC at a concentration of 3 × 10^5^ per well using UV-inactivated mpox and vaccinia. Figure 5A shows results on pre- and post-vaccination PBMC from three volunteers. UV-inactivated mpox at an MOI of 0.2 and UV-inactivated vaccinia at an MOI of 1 induced a markedly high number of SFCs compared to the corresponding stimulation conditions of pre-vaccination PBMC.

### 3.6. Testing Selected MOIs on Paired Samples

Selected MOIs of UV-inactivated mpox (MOI 0.2) and UV-inactivated vaccinia (MOI 1) viruses were tested on pre- and post-vaccination PBMC from seven volunteers. PBMC was used at a concentration of 3 × 10^5^/well and stimulated with UV-inactivated virus for 24 h. Figure 5B showed that the number of SFC in post-vaccination PBMC stimulated with UV-inactivated vaccinia was significantly higher compared to the corresponding SFC in pre-vaccination samples (mean ± SE of 26.3 ± 11.2 vs. 126.7 ± 46.4 with *p* = 0.03, Wilcoxon matched-pairs test). The number of SFC in post-vaccination PBMC stimulated with UV-inactivated mpox was higher compared to the corresponding SFC in pre-vaccination samples but did not reach statistical significance (mean ± SE of 11.4 ± 4.7 vs. 62.5 ± 31.2 with *p* = 0.063, Wilcoxon matched-pairs test). This is likely due to the sample size. When results obtained during initial testing of UV-inactivated mpox (Figure 5A) are included in the analysis (i.e., mean SFC of 3, 15, 5 in pre-vaccination samples from 3 volunteers vs. mean SFC of 300, 38, 12 in the corresponding post-vaccination samples stimulated with UV-inactivated mpox at MOI of 0.2), the post-vaccination SFC with mpox was significantly higher than the pre-vaccination SFC (*p* = 0.0078, Wilcoxon matched-pairs test). The number of SFC in pre-vaccination PBMC stimulated with mpox or vaccinia was not significantly different from SFC in the medium-rested control (*p* > 0.05). The number of SFC in post-vaccination PBMC stimulated with mpox or vaccinia was significantly higher compared to SFC in the medium-rested control (*p* < 0.05).

## 4. Discussion

Different new methods have been developed, including the use of recombinant vaccinia (e.g., expressing beta-galactosidase, GFP), but these assays suffer from most of the disadvantages of PRNT and are not useful for measuring mpox neutralizing antibody titer [30,31,32]. Microneutralization assays by performing the classic PRNT in 96-well plates have been developed for vaccinia and mpox but had turnaround times of 4–7 days [33,34,35,36]. Therefore, our results with this novel monoclonal antibody-based FRNT that could be conducted in a 96-well plate with a turnaround time of 2 days make detection of mpox neutralizing antibody titers faster. The turnaround time could be shortened further by decreasing the overnight incubation of the serum–virus mixture to 1 h. In fact, experiments on a few numbers of paired samples (*n* = 5) with 1 h versus overnight incubation of serum–virus mixtures showed similar FRNT results. Based on previous studies by our group and others using vaccinia, we believe that neutralization is likely IgG-mediated [37,38]. Identifying subtypes of IgG responsible for mpox neutralization is important. Poxviruses have two infectious forms: intracellular mature virion (IMV) and extracellular enveloped virion (EEV). EEV represents less than 1% of infectious progeny [39]. The proportion of EEV may decrease with further manipulation of viral culture (e.g., sonication) [40]. In our study, we did not identify specific neutralizing responses to IMV or EEV.

PRNT50, PRNT60, and PRNT90 have been used as a measure of smallpox vaccine-induced responses [14,24,25,29,41,42]. Our results showed that PRNT90 and FRNT90 were not reliable in identifying mpox-neutralizing antibody responses post-MVA-BN vaccination. FRNT50 and FRNT60 provided significantly higher titers in post-vaccination samples compared to pre-vaccination samples. These results were similar to results obtained by PRNT50 and PRNT60. The levels of FRNT50 or PRNT50 obtained in our post-vaccination samples were much higher than what was reported by others [11,12,43]. The main reason for this difference could be the timing of sample collection post-vaccination, which was 14 days after the last dose in our study and 3–9 months post-dose in the other studies. In our study, we compared neutralizing titers between pre- and post-vaccination matched samples. In studies without paired samples, other groups have used a cut-off of >10 to consider results as positive [44]. The newly developed FRNT makes screening of sera easier. Its 96-well plate format, instead of the traditional 24-well plates in PRNT, allows the use of fewer reagents and scalability to test a larger number of samples at one time. In addition, the 96-well plate format of FRNT makes the method potentially useful for screening new drugs against pox viruses by using different drug dilutions in place of sera. The methodology is not as complicated as PRNT, and therefore, it is easier to train new laboratory personnel. In addition to mpox, the new method can also be adapted to test vaccines against animal pox viruses such as swinepox virus, which may cause high rates of illness in pigs [45], and the African Swine Fever (ASF), a related disease that is lethal to pigs. Both swinepox and ASV virus are harmless to humans but have a huge potential impact on the economy [46].

ELISPOT is a quantitative method for measuring relevant parameters of T-cell activation. The sensitivity of IFN-γ ELISPOT, with a detection limit of approximately 0.06%, allows the detection of low-frequency antigen-specific T cells that secrete IFN-γ, an effector cytokine [47]. In this assay, the number of individual T cells secreting a cytokine after stimulation with a specific antigen is quantified [47]. The number of spots increases proportionately with the strength of the immune response. The number of PBMCs used per well and antigen concentration are two key variables that may affect the results of the IFN-γ ELISPOT assay [48,49,50]. We tested two different concentrations of PBMC, different antigen preparations, and different antigen concentrations when we optimized the assay for the detection of mpox-specific T-cell response. The 3 × 10^5^ PBMC per well and antigen stimulation time used in our assay were also used by other groups [36,44]. UV-inactivated mpox was used for IFN-γ ELISPOT by others to detect MVA-BN-induced mpox immunity, but MOIs of mpox used for stimulation were not clear [36]. The different IFN-γ ELISPOT results with live, heat-inactivated, and UV-inactivated viruses in our study could be related to the nature of antigens and processing by the antigen-presenting cells. Heat and UV inactivation are among the commonly used methods [51]. Heat inactivation may alter viral proteins and may denature the viral genome, whereas UV inactivation primarily affects the viral genome but preserves the sequences [52]. Inactivated viruses remain immunogenic, but determining the optimal concentration of inactivated viruses is important when they are used in vitro in immunological assays [53].

Different groups have used different methods of interpreting IFN-γ ELISPOT results. In the previously published study, ≥10 SFC/10^6^ PBMC was considered a positive test [36]. Other cut-off values, such as ≥50 SFC/10^6^ PBMC, ≥mean + 2SD of SFC of unvaccinated individuals, and ≥mean + 3SD of negative control, were used by other groups to interpret results of individuals with recent vaccination or mpox infection [36,44,54]. None of our pre-vaccination samples and 6 of 7 post-vaccination samples were positive if 10 SFC/10^6^ PBMC was used as a cut-off. Using a cut-off ≥mean + 2 SD [44], none of the pre-vaccination samples were positive, whereas all post-vaccination samples were positive. Using a cut-off of ≥50 SFC per 10^6^ PBMC [54], none of the pre-vaccination samples were positive, but only 2 of the 7 post-vaccination samples were positive. These indicate that different cut-offs lead to variations in interpretations of IFN-γ ELISPOT results. In our interpretation of IFN-γ ELISPOT results, we did not rely on cut-offs. We compared the absolute numbers of IFN-γ SFC in pre-vaccination samples with the corresponding post-vaccination samples. When pre-vaccination and post-vaccination samples are available, comparing changes in the number of IFN-γ SFC post-vaccination could be reliable.

Vaccine-induced neutralizing antibodies are important for mpox protection. Nonhuman primates depleted of B cells lack protection against mpox [55]. Similarly, both animal and human studies have shown the importance of T cells for mpox protection, particularly protection against severe disease and mortality [56,57]. Therefore, FRNT and IFN-γ ELISPOT assays developed in this study measure immune responses relevant to mpox protection.

This study has limitations. The number of samples used for development and optimization of assays was small. To limit the effect of a small sample size, we used pre- and post-vaccination samples, and we tested the samples multiple times. Studying changes in mpox-neutralizing antibody titers and T cell responses in a larger number of samples longitudinally by extending the time period post-vaccination to a few years and comparing MVA-induced mpox immune responses to responses induced by replication-competent smallpox vaccine(s) will be important. This study was conducted using samples from mpox low-risk individuals, and therefore, we did not assess vaccine efficacy or mpox disease severity post-vaccination.

## 5. Conclusions

In conclusion, neutralizing antibody titers and T-cell responses have been considered possible markers of protection against infections caused by pox viruses. Recent outbreaks of mpox have increased interest in the development of robust assays that reliably measure vaccine- or infection-induced immunity. Our newly developed microneutralization test based on the A29 monoclonal antibody has a turnaround time of 2 days and an excellent correlation with PRNT. IFN-γ ELISPOT using UV-inactivated mpox and 3 × 10^5^ PBMC per well identifies MVA-BN-induced T cell response. These assays will be useful in future mpox vaccine studies. The results obtained with optimized assays clearly show that a booster dose of MVA-BN for individuals with a history of smallpox vaccination and two doses for vaccinia-naïve individuals induce mpox-specific neutralizing antibody responses and T-cell immunity.

## Data Availability

Research data are available from the corresponding author upon request.

## Supplementary Materials

The following supporting information can be downloaded at: https://www.mdpi.com/article/10.3390/vaccines13010027/s1.

## References

[1] Tajudeen Y.A., Oladipo H.J., Muili A.O., Ikebuaso J.G. (2023). Monkeypox: A review of a zoonotic disease of global public health concern. Health Promot. Perspect..

[2] Americo J.L., Earl P.L., Moss B. (2023). Virulence differences of mpox (monkeypox) virus clades I, IIa, and IIb.1 in a small animal model. Proc. Natl. Acad. Sci. USA.

[3] Likos A.M., Sammons S.A., Olson V.A., Frace A.M., Li Y., Olsen-Rasmussen M., Davidson W., Galloway R., Khristova M.L., Reynolds M.G. (2005). A tale of two clades: Monkeypox viruses. J. Gen. Virol..

[4] Breman J.G., Kalisa R., Steniowski M.V., Zanotto E., Gromyko A.I., Arita I. (1980). Human monkeypox, 1970–1979. Bull. World Health Organ..

[5] Foster S.O., Brink E.W., Hutchins D.L., Pifer J.M., Lourie B., Moser C.R., Cummings E.C., Kuteyi O.E., Eke R.E.A., Titus J.B. (1972). Human monkeypox. Bull. World Health Organ..

[6] Yinka-Ogunleye A., Aruna O., Dalhat M., Ogoina D., McCollum A., Disu Y., Mamadu I., Akinpelu A., Ahmad A., Burga J. (2019). Outbreak of human monkeypox in Nigeria in 2017-18: A clinical and epidemiological report. Lancet Infect. Dis..

[7] Reynolds M.G., Davidson W.B., Curns A.T., Conover C.S., Huhn G., Davis J.P., Wegner M., Croft D.R., Newman A., Obiesie N.N. (2007). Spectrum of infection and risk factors for human monkeypox, United States, 2003. Emerg. Infect. Dis..

[8] Reed K.D., Melski J.W., Graham M.B., Regnery R.L., Sotir M.J., Wegner M.V., Kazmierczak J.J., Stratman E.J., Li Y., Fairley J.A. (2004). The detection of monkeypox in humans in the Western Hemisphere. N. Engl. J. Med..

[9] WHO 2022–2024 Mpox (Monkeypox) Outbreak: Global Trends. https://worldhealthorg.shinyapps.io/mpx_global/.

[10] Africa-CDC (2024). Event Based Surveillance Report, March 2024. Addis Ababa: ACDC. https://africacdc.org/download/africa-cdc-weekly-event-based-surveillance-report.

[11] Zaeck L.M., Lamers M.M., Verstrepen B.E., Bestebroer T.M., Van Royen M.E., Götz H., Shamier M.C., van Leeuwen L.P.M., Schmitz K.S., Alblas K. (2023). Low levels of monkeypox virus-neutralizing antibodies after MVA-BN vaccination in healthy individuals. Nat. Med..

[12] Moschetta N., Raccagni A.R., Bianchi M., Diotallevi S., Lolatto R., Candela C., Foppa C.U., Gismondo M.R., Castagna A., Nozza S. (2023). Mpox neutralising antibodies at 6 months from mpox infection or MVA-BN vaccination: A comparative analysis. Lancet Infect. Dis..

[13] Damon I.K., Davidson W.B., Hughes C.M., Olson V.A., Smith S.K., Holman R.C., Frey S.E., Newman F., Belshe R.B., Yan L. (2009). Evaluation of smallpox vaccines using variola neutralization. J. Gen. Virol..

[14] Newman F.K., Frey S.E., Blevins T.P., Mandava M., Bonifacio A., Yan L., Belshe R.B. (2003). Improved assay to detect neutralizing antibody following vaccination with diluted or undiluted vaccinia (Dryvax) vaccine. J. Clin. Microbiol..

[15] Katz J.B. (1987). The effect of the virus-serum incubation period upon vaccinia virus serum neutralization titers. J. Biol. Stand..

[16] Stienlauf S., Shoresh M., Solomon A., Lublin-Tennenbaum T., Atsmon Y., Meirovich Y., Katz E. (1999). Kinetics of formation of neutralizing antibodies against vaccinia virus following re-vaccination. Vaccine.

[17] Lansiaux E., Jain N., Laivacuma S., Reinis A. (2022). The virology of human monkeypox virus (hMPXV): A brief overview. Virus Res..

[18] Sagdat K., Batyrkhan A., Kanayeva D. (2024). Exploring monkeypox virus proteins and rapid detection techniques. Front. Cell Infect. Microbiol..

[19] Shchelkunov S., Totmenin A., Safronov P., Mikheev M., Gutorov V., Ryazankina O., Petrov N., Babkin I., Uvarova E., Sandakhchiev L. (2002). Analysis of the monkeypox virus genome. Virology.

[20] Franceschi V., Parker S., Jacca S., Crump R.W., Doronin K., Hembrador E., Pompilio D., Tebaldi G., Estep R.D., Wong S.W. (2015). BoHV-4-Based Vector Single Heterologous Antigen Delivery Protects STAT1(-/-) Mice from Monkeypoxvirus Lethal Challenge. PLoS Negl. Trop. Dis..

[21] Cai J.-P., Chu W.-M., Tam A.R., Wang K., Han Y., Chen L.-L., Zhang X., Choi C.Y.-K., Cheng V.C.-C., Chan K.-H. (2023). Determination of seroprevalence and kinetics of humoral response using mpox virus A29 protein. Commun. Med..

[22] von Krempelhuber A., Vollmar J., Pokorny R., Rapp P., Wulff N., Petzold B., Handley A., Mateo L., Siersbol H., Kollaritsch H. (2010). A randomized, double-blind, dose-finding Phase II study to evaluate immunogenicity and safety of the third generation smallpox vaccine candidate IMVAMUNE. Vaccine.

[23] Frey S.E., Wald A., Edupuganti S., Jackson L.A., Stapleton J.T., El Sahly H., El-Kamary S.S., Edwards K., Keyserling H., Winokur P. (2015). Comparison of lyophilized versus liquid modified vaccinia Ankara (MVA) formulations and subcutaneous versus intradermal routes of administration in healthy vaccinia-naive subjects. Vaccine.

[24] Frey S.E., Newman F.K., Kennedy J.S., Sobek V., Ennis F.A., Hill H., Yan L.K., Chaplin P., Vollmar J., Chaitman B.R. (2007). Clinical and immunologic responses to multiple doses of IMVAMUNE (Modified Vaccinia Ankara) followed by Dryvax challenge. Vaccine.

[25] Belshe R.B., Newman F.K., Frey S.E., Couch R.B., Treanor J.J., Tacket C.O., Yan L. (2004). Dose-dependent neutralizing-antibody responses to vaccinia. J. Infect. Dis..

[26] Frey S.E., Newman F.K., Kennedy J.S., Ennis F., Abate G., Hoft D.F., Monath T.P. (2009). Comparison of the safety and immunogenicity of ACAM1000, ACAM2000 and Dryvax in healthy vaccinia-naive adults. Vaccine.

[27] Talbot T.R., Stapleton J.T., Brady R.C., Winokur P.L., Bernstein D.I., Germanson T., Yoder S.M., Rock M.T., Crowe J.E., Edwards K.M. (2004). Vaccination success rate and reaction profile with diluted and undiluted smallpox vaccine: A randomized controlled trial. JAMA.

[28] Kennedy J.S., Gurwith M., Dekker C.L., Frey S.E., Edwards K.M., Kenner J., Greenberg R.N. (2011). Safety and immunogenicity of LC16m8, an attenuated smallpox vaccine in vaccinia-naive adults. J. Infect. Dis..

[29] Mazzotta V., Lepri A.C., Matusali G., Cimini E., Piselli P., Aguglia C., Lanini S., Colavita F., Notari S., Oliva A. (2024). Immunogenicity and reactogenicity of modified vaccinia Ankara pre-exposure vaccination against mpox according to previous smallpox vaccine exposure and HIV infection: Prospective cohort study. EClinicalMedicine.

[30] Manischewitz J., King L.R., Bleckwenn N.A., Shiloach J., Taffs R., Merchlinsky M., Eller N., Mikolajczyk M.G., Clanton D.J., Monath T. (2003). Development of a novel vaccinia-neutralization assay based on reporter-gene expression. J. Infect. Dis..

[31] Cosma A., Bühler S., Nagaraj R., Staib C., Hammarin A.-L., Wahren B., Goebel F.D., Erfle V., Sutter G. (2004). Neutralization assay using a modified vaccinia virus Ankara vector expressing the green fluorescent protein is a high-throughput method to monitor the humoral immune response against vaccinia virus. Clin. Diagn. Lab. Immunol..

[32] Earl P.L., Americo J.L., Moss B. (2003). Development and use of a vaccinia virus neutralization assay based on flow cytometric detection of green fluorescent protein. J. Virol..

[33] Manenti A., Solfanelli N., Cantaloni P., Mazzini L., Leonardi M., Benincasa L., Piccini G., Marchi S., Boncioli M., Raffaelli C.S. (2023). Evaluation of Monkeypox- and Vaccinia virus-neutralizing antibodies in human serum samples after vaccination and natural infection. Front. Public. Health.

[34] Grossegesse M., Stern D., Hofmann N., Surtees R., Kohl C., Michel J., Nitsche A. (2023). Serological methods for the detection of antibodies against monkeypox virus applicable for laboratories with different biosafety levels. J. Med. Virol..

[35] Grüner E., Grossegesse M., Stern D., Ober V., Eser T.M., Reiling G., Roider J. (2024). Mpox-specific immune responses elicited by vaccination or infection in people living with HIV. J. Infect. Dis..

[36] Sammartino J.C., Cassaniti I., Ferrari A., Piralla A., Bergami F., Arena F.A., Paolucci S., Rovida F., Lilleri D., Percivalle E. (2023). Characterization of immune response against monkeypox virus in cohorts of infected patients, historic and newly vaccinated subjects. J. Med. Virol..

[37] Lawrence S.J., Lottenbach K.R., Newman F.K., Buller RM L., Bellone C.J., Chen J.J., Cohen G.H., Eisenberg R.J., Belshe R.B., Stanley S.L. (2007). Antibody responses to vaccinia membrane proteins after smallpox vaccination. J. Infect. Dis..

[38] Pittman P.R., Hahn M., Lee H.S., Koca C., Samy N., Schmidt D., Hornung J., Weidenthaler H., Heery C.R., Meyer T.P. (2019). Phase 3 Efficacy Trial of Modified Vaccinia Ankara as a Vaccine against Smallpox. N. Engl. J. Med..

[39] Smith G.L., Vanderplasschen A. (1998). Extracellular Enveloped Vaccinia Virus.

[40] Locker J.K., Kuehn A., Schleich S., Rutter G., Hohenberg H., Wepf R., Griffiths G. (2000). Entry of the two infectious forms of vaccinia virus at the plasma membane is signaling-dependent for the IMV but not the EEV. Mol. Biol. Cell.

[41] Bunge E.M., Hoet B., Chen L., Lienert F., Weidenthaler H., Baer L.R., Steffen R. (2022). The changing epidemiology of human monkeypox-A potential threat? A systematic review. PLoS Negl. Trop. Dis..

[42] Wiser I., Orr N., Smetana Z., Spungin-Bialik A., Mendelson E., Cohen D. (2011). Alternative immunological markers to document successful multiple smallpox revaccinations. Clin. Infect. Dis..

[43] Collier A.-R.Y., McMahan K., Jacob-Dolan C., Liu J., Borducchi E.N., Moss B., Barouch D.H. (2024). Decline of Mpox Antibody Responses After Modified Vaccinia Ankara-Bavarian Nordic Vaccination. JAMA.

[44] Matusali G., Petruccioli E., Cimini E., Colavita F., Bettini A., Tartaglia E., Maggi F. (2023). Evaluation of Cross-Immunity to the Mpox Virus Due to Historic Smallpox Vaccination. Vaccines.

[45] Medaglia M.L., Pereira Ade C., Freitas T.R., Damaso C.R. (2011). Swinepox virus outbreak, Brazil, 2011. Emerg. Infect. Dis..

[46] Ruedas-Torres I., Nga B.T.T., Salguero F.J. (2024). Pathogenicity and virulence of African swine fever virus. Virulence.

[47] Schlingmann T.R., Shive C.L., Targoni O.S., Tary-Lehmann M., Lehmann P.V. (2009). Increased per cell IFN-gamma productivity indicates recent in vivo activation of T cells. Cell Immunol..

[48] Karulin A.Y., Caspell R., Dittrich M., Lehmann P.V. (2015). Normal Distribution of CD8+ T-Cell-Derived ELISPOT Counts within Replicates Justifies the Reliance on Parametric Statistics for Identifying Positive Responses. Cells.

[49] Sundararaman S., Karulin A.Y., Ansari T., BenHamouda N., Gottwein J., Laxmanan S., Levine S.M., Loffredo J.T., McArdle S., Neudoerfl C. (2015). High Reproducibility of ELISPOT Counts from Nine Different Laboratories. Cells.

[50] Barabas S., Spindler T., Kiener R., Tonar C., Lugner T., Batzilla J., Bendfeldt H., Rascle A., Asbach B., Wagner R. (2017). An optimized IFN-gamma ELISpot assay for the sensitive and standardized monitoring of CMV protein-reactive effector cells of cell-mediated immunity. BMC Immunol..

[51] Patterson E.I., Prince T., Anderson E.R., Casas-Sanchez A., Smith S.L., Cansado-Utrilla C., Hughes G.L. (2020). Methods of Inactivation of SARS-CoV-2 for Downstream Biological Assays. J. Infect. Dis..

[52] Elveborg S., Monteil V.M., Mirazimi A. (2022). Methods of Inactivation of Highly Pathogenic Viruses for Molecular, Serology or Vaccine Development Purposes. Pathogens.

[53] Croft S., Wong Y.C., Smith S.A., Flesch I.E.A., Tscharke D.C. (2020). Surprisingly Effective Priming of CD8(+) T Cells by Heat-Inactivated Vaccinia Virus Virions. J. Virol..

[54] Moraes-Cardoso I., Benet S., Carabelli J., Perez-Zsolt D., Mendoza A., Rivero A., Alemany A., Descalzo V., Alarcón-Soto Y., Grifoni A. (2024). Immune responses associated with mpox viral clearance in men with and without HIV in Spain: A multisite, observational, prospective cohort study. Lancet Microbe.

[55] Edghill-Smith Y., Golding H., Manischewitz J., King L.R., Scott D., Bray M., Nalca A., Hooper J.W., Whitehouse C.A., Schmitz J.E. (2005). Smallpox vaccine-induced antibodies are necessary and sufficient for protection against monkeypox virus. Nat. Med..

[56] Smith Y.E., Bray M., Whitehouse C.A., Miller D., Mucker E., Manischewitz J., King L.R., Guroff M.R., Hryniewicz A., Venzon D. (2005). Smallpox vaccine does not protect macaques with AIDS from a lethal monkeypox virus challenge. J. Infect. Dis..

[57] Taha A.M., Elrosasy A., Mahmoud A.M., Saed S.A.A., Moawad W.A.E., Hamouda E., Nguyen D., Tran V.P., Pham H.T., Sah S. (2024). The effect of HIV and mpox co-infection on clinical outcomes: Systematic review and meta-analysis. HIV Med..

