# Supplementary material for: Optimizing Microneutralization and IFN-γ ELISPOT Assays to Evaluate Mpox Immunity
Source: Vaccines (Basel). 2024 Dec 31;13(1):27. doi: 10.3390/vaccines13010027 (PMC11769350; doi:10.3390/vaccines13010027)
Supplement: Supplementary file 1 [file vaccines-13-00027-s001.zip › vaccines-3359229-supplementary.pdf]

Supplementary Table S1. Materials and Reagents for FRNT assay.

**Materials and reagents**	**Sources**
Mpox virus	Bei resources, NR-2500
Purified buffer saline (PBS)	Fisher SH30256.02
Fetal bovine serum (FBS)	Sigma
BSC40 cells	ATCC
96-well plate	Fisher 07-200-90
Filtered pipet tips	MidSci, PR-RK-FL
O-ring sealed 2ml tubes	Sarstedt
2% Carboxymethylcellulose	Sigma Cat M0512-250G
MPXV A29 monoclonal antibody	Sino Biological 40891-M0017
Secondary Antibody 1:2000 anti-mouse HRP-label antibody	Invitrogen G-21040
Peroxidase substrate	Trublue Peroxidase Substrate – 5510-0030
Bleach	Fisher NC9724348
Oxivir TB Disinfectant	Amazon
DMEM	Sigma D5796-500ml
Flask	Fisher 10-126-34
HEPES	Fisher 15-630-080
Tripsin-EDTA	Fisher 25-200-056
Penicillin-Streptomycin	Fisher 15-140-122
L-Glutamine	Fisher 25-030-81
20% Paraformaldehyde	VWR AA47340-9M
Triton X-100	Sigma X100-500ml
Saponin	Sigma 47036-50g-F
10XPBS	Fisher BP3994
1X Focus forming assay (FFA) Wash Buffer	1L 10X PBS + 9L Millipore H_20 + 5ml Triton X-100
5X FFA Staining Buffer	250 ml 10X PBS, 250 ml Millipore H_2O and 2.5 grams saponin
Growth Media (5% DMEM)	500 ml DMEM+ 5ml 1M HEPES solution + 25ml FBS
5% Paraformaldehyde (PFA	2.5ml 20% PFA + 7.5ml 1X PBS
Primary A29 Antibody (Sino Biological)	2 ml 5X FFA Staining Buffer + 8 ml Millipore H_2O +2 µl Primary Antibody
Secondary Goat Anti-mouse Antibody (Invitrogen)	2 ml 5X FFA Staining Buffer + 8 ml Millipore H_2O +5 µl Primary Antibody
Dry shoe covers	Thomas Scientific 20A00D017

Materials and reagents	Sources
Liquid impermeable shoe covers	VWR 414004-641
Isolation gowns	Thomas Scientific 20A00M242
Liquid impermeable gowns	Fisher 18-567
N95 Face masks	Fisher 19-164-104
Disposable face shields	Fisher 17-310

Supplementary Table S2. Materials and reagents for IFN-γ ELISPOT assay.

Materials and Reagents	Source
Phosphate Buffered Saline (PBS)	FisherSci SH30256.02
0.05% Tween 20 in PBS	Fisher AC233362500
FBS	Sigma F2442
Sodium Pyruvate	FisherSci 11360-070
RPMI +3 with FBS	RPMI (Life Tech 11875-093)<br>L-Glutamine 200nM (Fisher #:25-030-081)<br>Penicillin/Streptomycin<br>(FisherSci #15-140-122)
Substrate solution	3-amino-9-ethyl-carbazole (AEC) (Sigma A-5754)<br>Dimethyl formamide (DMF) (Sigma D-4551)<br>Acetic acid/glacial acetic acid (Sigma Cat # A38-212)<br>Sodium acetate (Sigma Cat# S8625)<br>Hydrogen peroxide (Sigma H1009)
Dilution Buffer	See PBS and RPMI
Elispot Kit with some reagents	BD Bioscience<br>551849
10% formalin	FisherSci 305-510
DI H_2O	FisherSci: 5H3052903
Bleach	FisherSci NC9724348
Oxivir TB Disinfectant	Amazon 4277285
Mpox (live)	Bei resources NR-2500
Vaccinia (live)	Dryvax
PMA	Sigma<br>Cat No: P8139
Gloves	FisherSci 19-177-522
Pipettes	FischerSci<br>13-678-11C/2ml

**Materials and Reagents**	**Source**
	Costar<br>4487/5ml,<br>Costar 4488/10ml Costar 4489/25ml
Dry shoe covers	Thomas Scientific<br>20A00D017
Liquid impermeable shoe covers	VWR<br>414004-641
Isolation gowns	Thomas Scientific<br>20A00M242
Liquid impermeable gowns	FisherSci<br>18-567
N95 Face masks	FisherSci<br>19-164-104
Disposable face shields	FisherSci<br>17-310
Barrier pipette tips	MidSci<br>PR-1250RK-FL, PR-10RK-FL, PR-20RK-FL, PR-200RK-FL
Centrifuge tubes	FisherSci<br>50 ml: 14-959-49<br>15 ml: 05-539-5
Bottle top filters	FisherSci SCGPS02RE/250ml SCGPS05RE/500ml
Syringe filters	FisherSci SLGPO33RS
Barrier pipette Tips	MidSci PR-1250RK-FL, PR-10RK-FL, PR-20RK-FL, PR-200RK-FL
10 ml Syringes	FisherSci 14955495
Alcohol swab	FisherSci 13-680-63
Kimtech Precision Wipes	FisherSci 06-677-73
Disposable Reagent Reservoirs	Fisher 07-200-128; 077-200-129

Supplementary Figure S1.

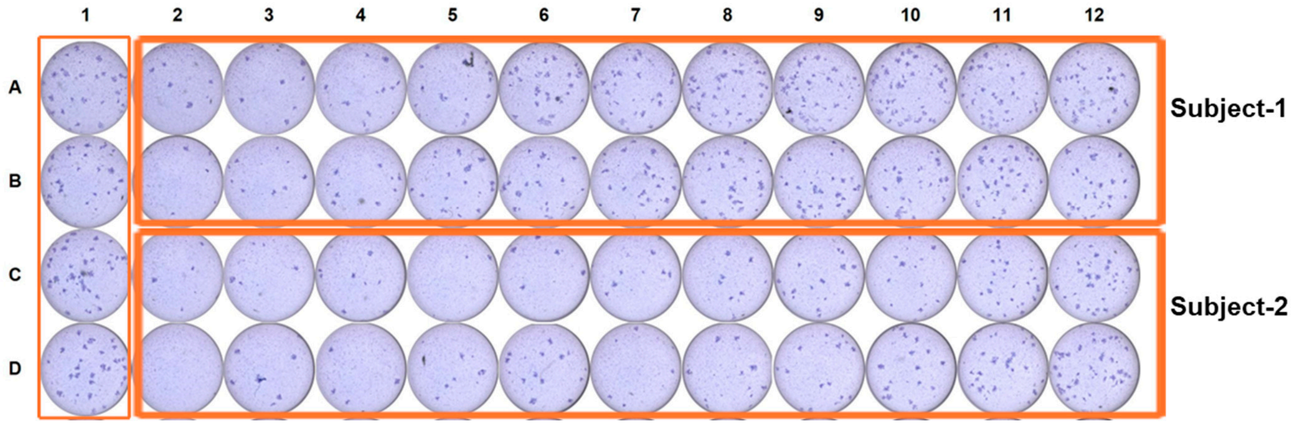

Supplementary Figure S1. Typical focus forming units in mpox FRNT. Half of the 96-well plate is shown. Wells A1, B1, C1 and D1 contain serum-free virus (i.e., control). The first 2 rows are for sample-1 and the next two rows are for sample-2 tested in duplicate. Double dilution of sera from the two subjects were diluted starting from 1 in 4 in 2A and 2B for sample-1, and 2C and 2D. The last wells contain 1:4096 dilution of sera. Spots are focus forming units of mpox detected using A29 monoclonal antibody.

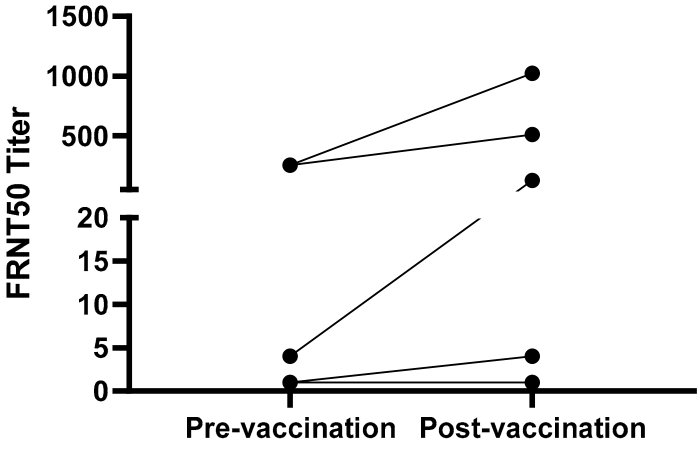

Supplementary Figure S2. Vaccinia FRNT. A27, a monoclonal antibody for vaccinia was tested as a primary antibody in the FRNT. The results showed FRNT50 results on pre-and post-vaccination samples (n=6). Pre-and post-vaccination samples had FRNT50 of 86.5 ± 53.6 and 283.5 ± 167.9 (Mean ± SE), respectively. Two samples with higher pre-vaccination FRNT50 were from volunteers with history of prior smallpox vaccination.
